# Supplementary figures and images for: Computational Analysis of Short Linear Motifs in the Spike Protein of SARS-CoV-2 Variants Provides Possible Clues into the Immune Hijack and Evasion Mechanisms of Omicron Variant
Source: Int J Mol Sci. 2022 Aug 8;23(15):8822. doi: 10.3390/ijms23158822 (PMC9368778; doi:10.3390/ijms23158822)

# Supplementary Figure S1

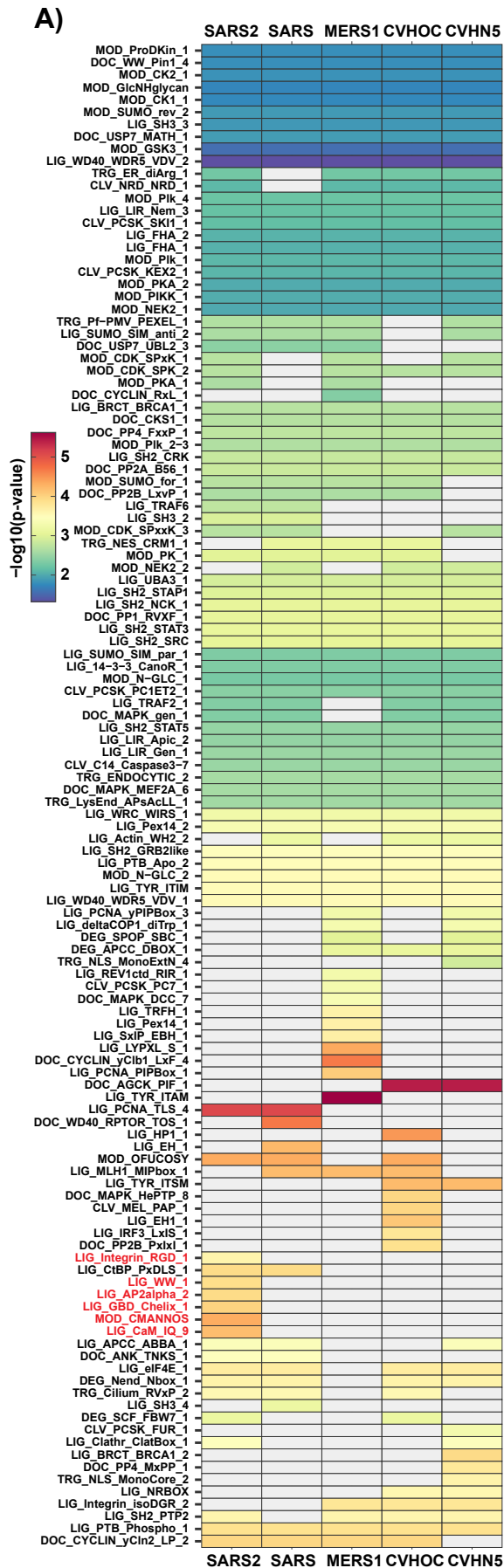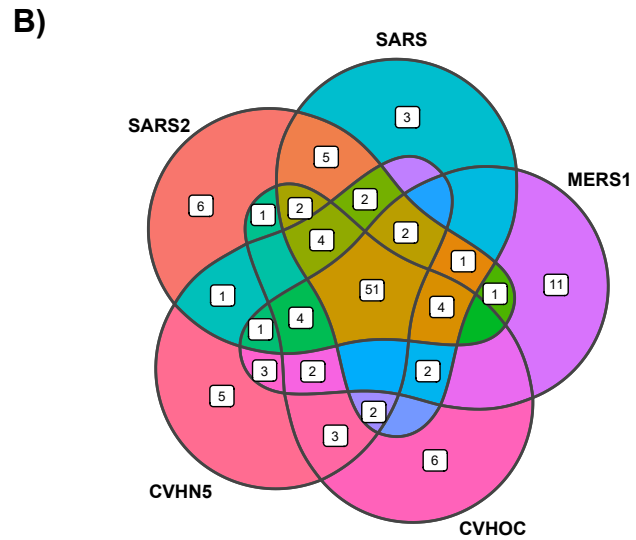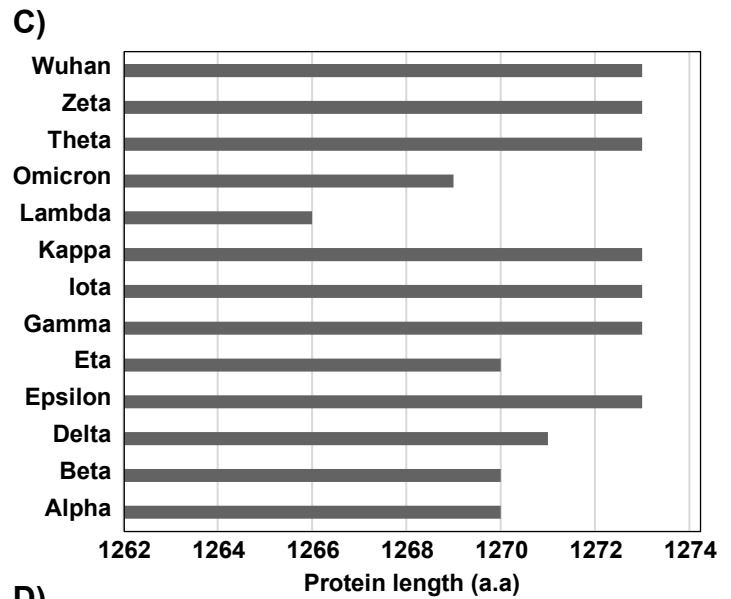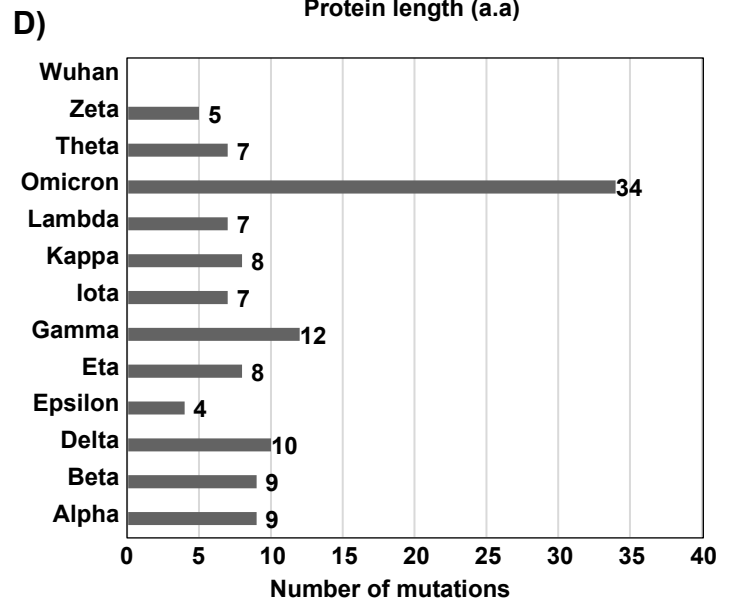

Supplement: Supplementary file 1 [file ijms-23-08822-s001.zip › ijms-1798083-supplementary.pdf]
